# Alterations in the gut microbiota and serum metabolomics of spontaneous cholestasis caused by loss of FXR signal in mice

**DOI:** 10.3389/fphar.2023.1197847

**Published:** 2023-05-22

**Authors:** Shizhang Wei, Tingting He, Xu Zhao, Manyi Jing, Haotian Li, Lisheng Chen, Ruimao Zheng, Yanling Zhao

**Affiliations:** ^1^ Department of Anatomy, Histology and Embryology, School of Basic Medical Sciences, Peking University, Beijing, China; ^2^ Department of Pharmacy, Chinese PLA General Hospital, Beijing, China; ^3^ Division of Integrative Medicine, The Fifth Medical Center, Chinese PLA General Hospital, Beijing, China

**Keywords:** cholestasis, microbiome, metabolomics, bile acid, FXR

## Abstract

**Background:** Farnesoid X receptor (FXR) is a key metabolic target of bile acids (BAs) and is also a target for drugs against several liver diseases. However, the contribution of FXR in the pathogenesis of cholestasis is still not fully understood. The purpose of this study is to provide a comprehensive insight into the metabolic properties of FXR-involved cholestasis in mice.

**Materials and methods:** In this study, an alpha-naphthylisothiocyanate (ANIT)-induced cholestasis mouse model and FXR^−/−^ mice were established to investigate the effect of FXR on cholestasis. The effect of FXR on liver and ileal pathology was evaluated. Simultaneously, Untargeted metabolomics combined with 16s rRNA gene sequencing analysis was applied to reveal the involvement of FXR in the pathogenesis of cholestasis.

**Results:** The results showed that ANIT (75 mg/kg) induced marked cholestasis in WT and FXR −/− mice. It is noteworthy that FXR^−/−^ mice developed spontaneous cholestasis. Compared with WT mice, significant liver and ileal tissue damage were found. In addition, 16s rRNA gene sequencing analysis revealed gut microbiota dysbiosis in FXR−/− mice and ANIT-induced cholestasis mice. Differential biomarkers associated with the pathogenesis of cholestasis caused by FXR knockout were screened using untargeted metabolomics. Notably, *Lactobacillus*_ *johnsonii*_FI9785 has a high correlation with the differential biomarkers associated with the pathogenesis and progression of cholestasis caused by FXR knockout.

**Conclusion:** Our results implied that the disorder of the intestinal flora caused by FXR knockout can also interfere with the metabolism. This study provides novel insights into the FXR-related mechanisms of cholestasis.

## 1 Introduction

Disorder of bile acid metabolism is the most common cause of many liver diseases. FXR is a protective sensor that induces protective cellular responses in liver and gastrointestinal tissues and is involved in mediating inflammatory responses, immune responses, and liver regeneration (Lefebvre et al., 2009; Gadaleta et al., 2011). More importantly, FXR is a key nuclear receptor for bile acid metabolism (Allen et al., 2011; Wang et al., 2012; Chiang, 2013). Unconjugated bile acids can downregulate downstream proteins ASBT, IBABP, and OST-α/β by activating FXR in the ileum (Gadaleta et al., 2015). Sodium-dependent taurocholic acid co-transport peptide (NTCP) is mainly responsible for the hepatic uptake of conjugated bile acids (80%). Ileal bile acid is mainly absorbed by NTCP after being circulated to the liver (Kullak-Ublick et al., 2004). NTCP is negatively regulated by nuclear FXR through a feedback mechanism to limit the increase of hepatic bile acid levels (Matsubara et al., 2013). In the case of cholestasis, FXR induces the expression of OST-α/β in the sinus membrane to promote the inflow of bile acid into the systemic circulation (Boyer et al., 2006; Landrier et al., 2006). In mice, taurine-advanced *β* MCA (T-βMCA) antagonizes FXR signals in the intestine. T-βMCA can induce FGFR4-dependent activation of CYP7A1 via inhibition of JUN signals and causes the liver to synthesize more bile acid. Intestinal microbiota deconjugate T-βMCA and promotes FXR signal transduction. However, T-βMCA cannot be metabolized without bacteria (Stanimirov et al., 2012; Sayin et al., 2013).

The human gastrointestinal tract is colonized by various symbiotic bacteria and other microorganisms (Nicholson et al., 2012). The host genome and microbiome jointly produce a large number of metabolites that can participate in the metabolic process as important signal factors and energy substrates in the body, such as the bile acids that participated in the digestive process or pathogenic process (Monte et al., 2009; Musso et al., 2011; Swann et al., 2011; Correa-Oliveira et al., 2016). The dynamic characteristics of these intestinal metabolites participate in the regulation of metabolic phenotype (Iida et al., 2013; Zheng et al., 2013; Jia et al., 2018). Intestinal flora can convert intestinal bile acid into unconjugated bile acids and activates bile acid receptors, such as FXR, G protein-coupled receptor 5 (TGR5), pregnane X receptor (PXR), constitutive androstane receptor (CAR) and vitamin D receptor (VDR) (Thomas et al., 2008; Hylemon et al., 2009). There are many kinds of intestinal bacteria are also known to be involved in the metabolism of bile acids (BAs): *Bacteroides, Listeria*, *Lactobacillus*, *Ruminococcus*, *Bifidobacterium*, *Eubacterium*, *Escherichia*, *Egghertella*, *Peptostreptococcus* and *Clostridium* (Chiang, 2009; Gerard, 2013). In general, intestinal bacteria and FXR play a key role in the enterohepatic circulation of bile acid.

In this study, we intend to reveal the influence of FXR on the metabolic characteristics of the body and the difference in intestinal bacteria by applying metabolomics and intestinal bacteria analysis methods based on the perspective of the “gut-liver axis.”

## 2 Materials and methods

### 2.1 Experimental animals

Twelve male 8-week-old C57BL/6J mice and 12 female 8-week-old FXR^−/−^ mice were randomly divided into four groups of six mice: Wild-type control group (WT), FXR control group (FXR^−/−^), wild-type model group (WT + ANIT) and FXR model group (FXR^−/−^ + ANIT). The mice in WT and FXR^−/−^ groups were treated with vehicle (olive oil), and the mice in the WT + ANIT and FXR^−/−^ + ANIT groups were treated with ANIT (75 mg/kg) by oral gavage. After 48 h, the mice in each group were fasted for 12 h on the night prior to experimental sample collection. Blood samples (∼0.6 mL for each mouse) were collected from the infraorbital venous plexus of mice and were separated by centrifugation at 3,000 rpm for 10 min for untargeted metabolomics analysis. The ileum and the part of the large hepatic lobe were fixed with 10% formaldehyde for histopathological examination. The intestinal contents were collected and immediately snap-frozen in liquid nitrogen for 16s rRNA gene sequencing analysis. All animal experiments performed in this study were approved and supervised by the ethics committee of the fifth medical center of PLA general hospital (Beijing, China).

### 2.2 Serum biochemical assays

The serum concentrations of aspartate transaminase (AST), alanine transaminase (ALT), total bile acid (TBA), and direct bilirubin (DBIL) were measured according to the manufacturer’s protocols.

### 2.3 Pathologic analysis

The liver and ileum tissues of mice obtained from each group were fixed in 4% paraformaldehyde, embedded in paraffin, and stained with hematoxylin and eosin (H&E). Pathological photographs were obtained using a microscope (magnification ×200 and 400×). The hepatic HE scores were assessed according to previous research (Gong et al., 2021). Briefly, the hepatic histological parameters of inflammation, necrosis, and hepatocyte vacuolization were all assessed on a scale of 0–3 (0 defined as absent; 3 defined as severe). Chiu’s score was used to evaluate the level of ileum injury (Li et al., 2020).

### 2.4 Serum sample handling and metabolomics determination

To prepare samples for the untargeted metabolomics analysis, frozen serum samples were allowed to thaw at room temperature. 200 μL of each serum sample was mixed with 600 μL of 100% methanol and was then centrifuged for 10 min at 48,000 rpm. A total of 500 μL supernatant was transferred to a new 1.5 mL tube and dried with a nitrogen blower. The dried samples were reconstituted with 500 μL of 50% acetonitrile methanol. Dissolved samples were filtered into the sample bottle with a 0.22-μm membrane for Q-TOF/MS analysis as described previously (Wei et al., 2018).

### 2.5 Data extraction and multivariate analysis

Data analysis was performed within Mass Hunter Profinder software (Agilent) for post-acquisition data processing. Then, the MetaboAnalyst database was used to normalize the original data. Multivariate data analysis was performed with SIMCA 14.0 (Umetrics, Sweden). OPLS-DA analysis based on non-targeted metabolomics was conducted in the serums between WT and WT + ANIT group, and WT and FXR-KO group (n: WT = 6, WT + ANIT = 6, FXR^−/−^ = 6). The endogenous metabolites with VIP >1.5 and |p(corr)| ≥ 0.58 were selected as the differential biomarker for further pathway enrichment analysis. MetaboAnalyst and DAVID database were used to analyze the metabolic pathway and KEGG pathway. Metabolite targets were collected through the MBrole database (http://csbg.cnb.csic.es/mbrole2/) and applied to the Sangerbox database (http://sangerbox.com/) for GO enrichment analysis and the Metascape database (https://metascape.org/gp/) for protein interaction analysis.

### 2.6 16s rRNA sequencing

The total DNA of intestinal bacteria was extracted from intestinal contents using HiPure Stool DNA Kit B according to the manufacturer’s instructions. The DNA extractions were quantified by ultraviolet spectroscopy and amplified using universal primers of 341F: 5′-CCTACGGGNGGCWGCAG-3′ and 806R: 5′-GGACTACHVGGGTATCTAAT-3′ to target the V3–V4 region of bacterial 16s rRNA. The raw data obtained were then analyzed as described previously (Guo et al., 2017).

### 2.7 Other statistical analysis

Statistical analyses were performed with SPSS 19.0, All experimental data were expressed as the mean ± standard deviation. The differences between the group means were calculated by one-way analysis. *p* < 0.05 were considered statistically significant.

## 3 Results

### 3.1 FXR knockout results in spontaneous cholestasis in mice

**FIGURE 1:**
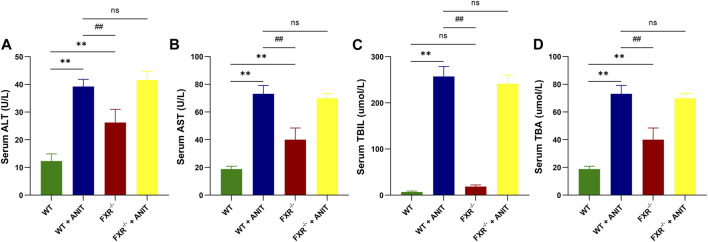
FXR-KO results in spontaneous cholestasis in mice. **(A)** Serum ALT level. **(B)** Serum AST level. **(C)** Serum TBIL level. **(D)** Serum TBA level. Data shown are the means ± SD. **p < 0.01 compared with the WT group; # *p* < 0.05, ## *p* < 0.01 compared with the WT + ANIT group.

As shown in Figure 1, ANIT caused significant increases in serum ALT, AST, TBIL, and TBA levels in WT and FXR^−/−^ mice. Compared with WT + ANIT mice, no significant change in serum ALT, AST, TBIL, and TBA levels in FXR^−/−^ + ANIT mice.

### 3.2 Effect of FXR knockout on liver and ileum pathology

**FIGURE 2:**
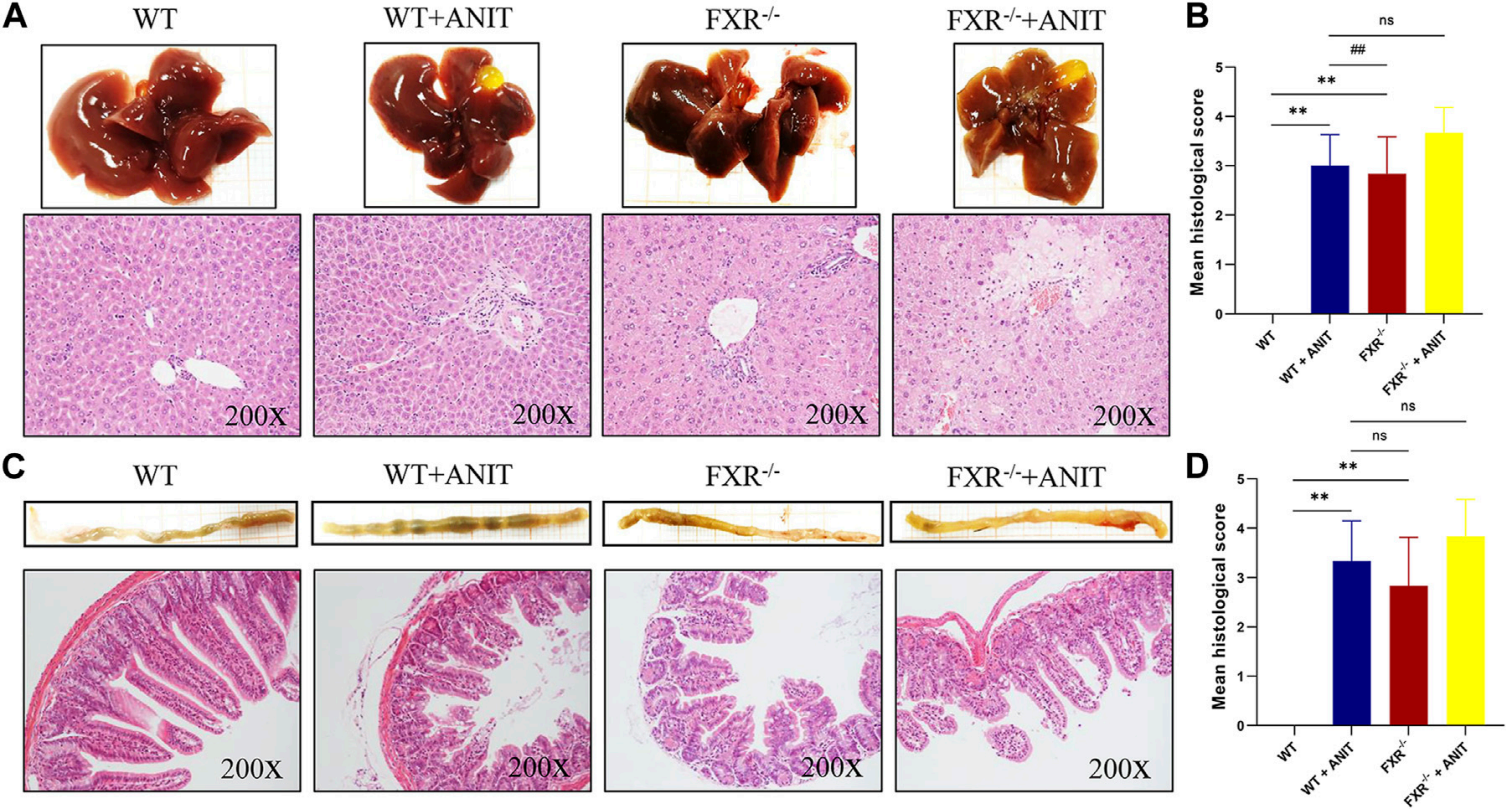
Effect of FXR on liver and ileum histopathology. **(A)** Overall image of the liver and representative image of HE staining (×200 magnification). **(B)** The liver histological score. **(C)** Overall image of the ileum and the representative image of HE staining (×200 magnification). **(D)** The ileum histological score. Data shown are the means ± SD. ** *p* < 0.01 compared with the WT group; ## *p* < 0.01 compared with the WT + ANIT group.

Histological analysis of the liver and ileum provided direct evidence of FXR function in cholestatic liver injury. As shown in Figures 2A, B, compared with WT mice, the WT + ANIT and FXR^−/−^ + ANIT mice showed enlarged gallbladders. The liver tissue of mice in WT + ANIT, FXR^−/−^, and FXR^−/−^ + ANIT groups showed marked acute infiltration, hepatic necrosis, and hepatocyte vacuolization. Pathological changes in the mouse ileum suggested that the ileum in the WT + ANIT, FXR^−/−^, and FXR^−/−^ + ANIT groups had a more intense yellow color compared with the mice in the WT group. And compared with the WT group, the mice in the WT + ANIT, FXR^−/−^ and FXR^−/−^ + ANIT groups had a shed, sparse, and low ileum villi combined with obvious inflammatory cell infiltration (Figures 2C, D).

### 3.3 Effect of FXR knockout on serum metabolic characteristics in mice

**FIGURE 3:**
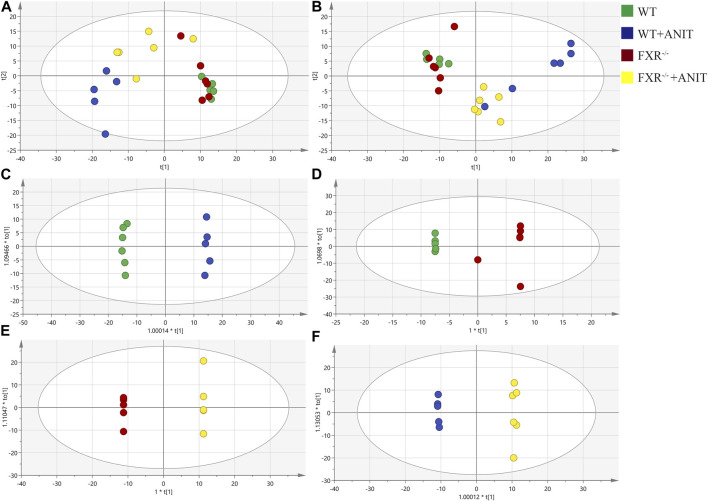
PCA and OPLS-DA analysis of serum metabolic data. **(A)** PCA score plot in positive-ion mode. **(B)** PCA score plot in negative-ion mode. OPLS-DA score chart of the serum metabolite analysis between the WT and WT + ANIT groups **(C)**, between the WT and FXR^−/−^ groups **(D)**, between the FXR^−/−^ and FXR^−/−^ + ANIT groups **(E)**, and between the WT + ANIT and FXR^−/−^ + ANIT groups **(F)**. The oval in the graph indicates the 95% confidence interval.

To discover the regulatory mechanism of FXR on the pathogenesis of intrahepatic cholestasis, the effect of FXR on serum metabolic microenvironment *in vivo* was studied by metabolomics, and the signal network regulated by FXR was further tracked by FXR-related specific biomarkers. The PCA results showed that four clusters could be better distinguished (Figures 3A, B). OPLS-DA analysis of metabolite profiles also showed a global metabolic difference between WT and WT + ANIT groups (R2X(cum) = 0.431, R2Y(cum) = 0.998, Q2(cum) = 0.956), WT and FXR^−/−^ groups (R2X(cum) = 0.658, R2Y(cum) = 0.833, Q2(cum) = 0.334), FXR^−/−^ and FXR^−/−^ + ANIT groups (R2X(cum) = 0.621, R2Y(cum) = 1, Q2(cum) = 0.825), WT + ANIT and FXR^−/−^ + ANIT groups (R2X(cum) = 0.505, R2Y(cum) = 0.999, Q2(cum) = 0.775) in positive-ion (ESI+) mode (Figures 3C–F). Meanwhile, the OPLS-DA analysis had similar results in negative-ion (ESI-) mode (Supplementary Figure S1).

### 3.4 Effect of FXR knockout on serum metabolites in mice with intrahepatic cholestasis

**FIGURE 4:**
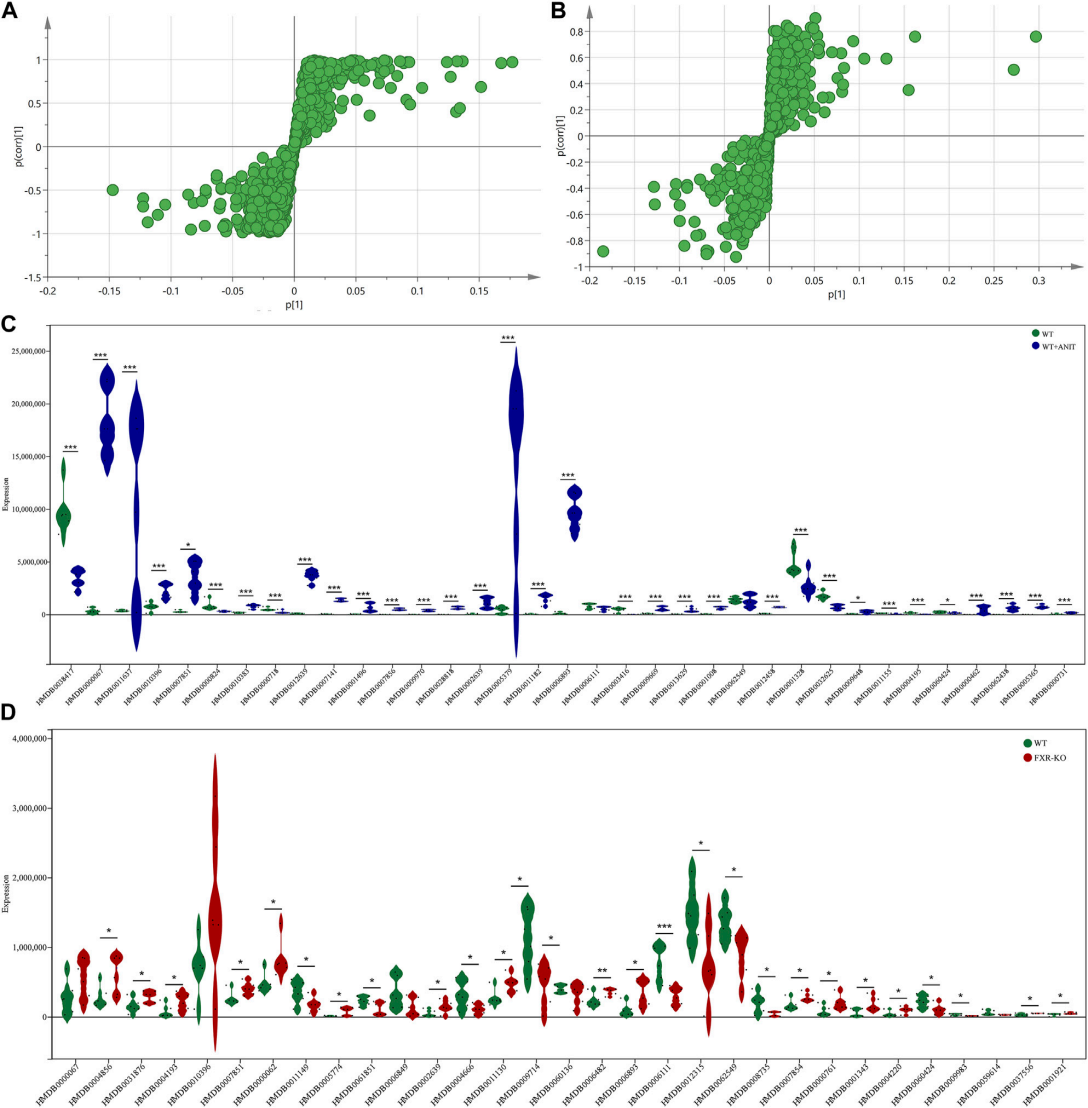
The scatter plots of serum metabolic data and the map displaying the expression of different biomarkers. The scatter plots of serum metabolic data of WT vs. WT + ANIT **(A)** and WT vs. FXR^−/−^ **(B)**. **(C)** The map of the expression of different biomarkers between the WT and WT + ANIT groups. **(D)** The map of the expression of different biomarkers between the WT and FXR^−/−^ groups. *p < 0.05; **p < 0.01; ***p < 0.001 compared with the corresponding control group.

To further analyze the disturbance of the serum biomarkers in mice with intrahepatic cholestasis caused by FXR knockout, we conducted an in-depth analysis of the OPLS-DA models of serum samples between the WT and WT + ANIT groups, and between the WT and FXR^−/−^ groups. A scatter analysis diagram (Figures 4A, B and Supplementary Figure S2) was established using p and p (corr) parameters. The metabolites with VIP>1 and |p(corr)|>0.58 were regarded as the differential biomarkers of the WT vs. WT + ANIT and WT vs. FXR^−/−^ respectively. 35 differential serum metabolites were found in both the positive-ion mode and the negative-ion mode. Compared with the WT group, the expression of the above 35 biomarkers in the WT + ANIT model was almost significant (Figure 4C). In the scatter analysis data of WT vs. FXR^−/−^, 31 differential serum biomarkers were found in both the positive-ion and the negative ion modes (Figure 4D).

**FIGURE 5:**
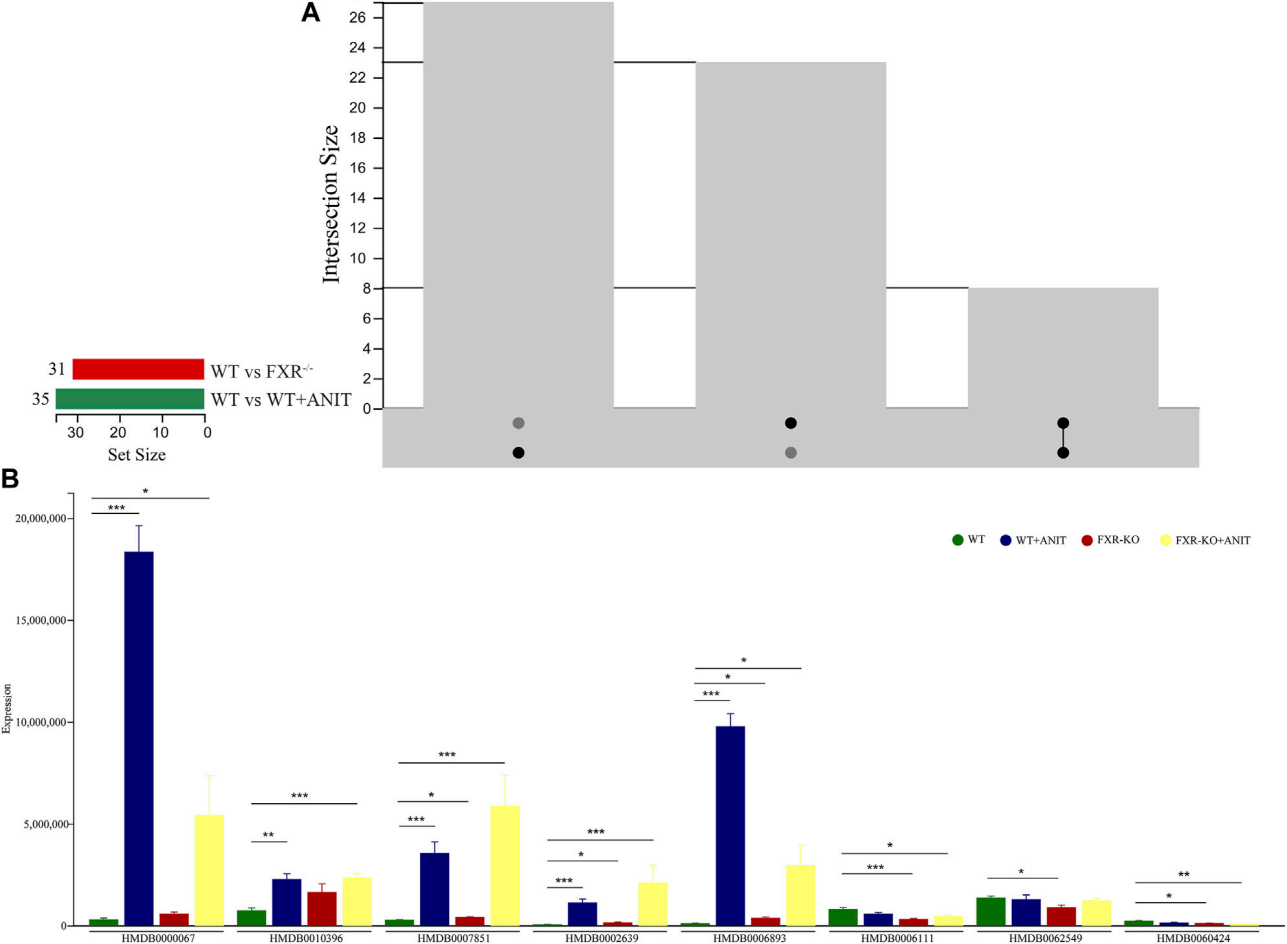
Screening of serum differential biomarkers associated with FXR deficiency-induced intrahepatic cholestasis. **(A)** Venn diagram of serum differential biomarkers of WT vs. WT + ANIT and WT vs. FXR^−/−^. **(B)** The expression of the serum differential biomarkers shared by WT vs. WT + ANIT and WT vs. FXR^−/−^. *p < 0.05; **p < 0.01; ***p < 0.001.

Several differential biomarkers triggered by FXR deficiency and intrahepatic cholestasis induced by ANIT in WT mice were obtained. Considering the function of FXR in many diseases, the differential biomarkers shared by WT vs. WT + ANIT and WT vs. FXR^−/−^ were further screened by using a Venn plotter to explore the specific biomarkers associated with the pathogenesis of cholestasis caused by FXR knockout (Figure 5A). To this end, 8 differential biomarkers were further screened in serum. (Figure 5B; Table 1).

**TABLE 1:** Serum differential biomarkers associated with FXR deficiency-induced intrahepatic cholestasis in mice.

No.	Hit	HMDB	PubChem	KEGG
1	7alpha-Hydroxypregnenolone	HMDB0060424	9,967,418	C18038
2	Cholesterol	HMDB0000067	5,997	C00187
3	LysoPC(20:4(8Z,11Z,14Z,17Z))	HMDB0010396	53,480,469	C04230
4	LysoPA(0:0/18:1(9Z))	HMDB0007851	56,947,016	C00416
5	Sulfolithocholylglycine	HMDB0002639	53,477,756	C11301
6	3a,7a-Dihydroxy-5b-cholestane	HMDB0006893	3,080,603	C05452
7	12-HETE	HMDB0006111	5,312,983	C14777
8	2-Hydroxystearic acid	HMDB0062549	439,887	C03045

### 3.5 Pathway enrichment analysis of serum differential biomarker and the target screening of FXR deficiency-induced intrahepatic cholestasis

**FIGURE 6:**
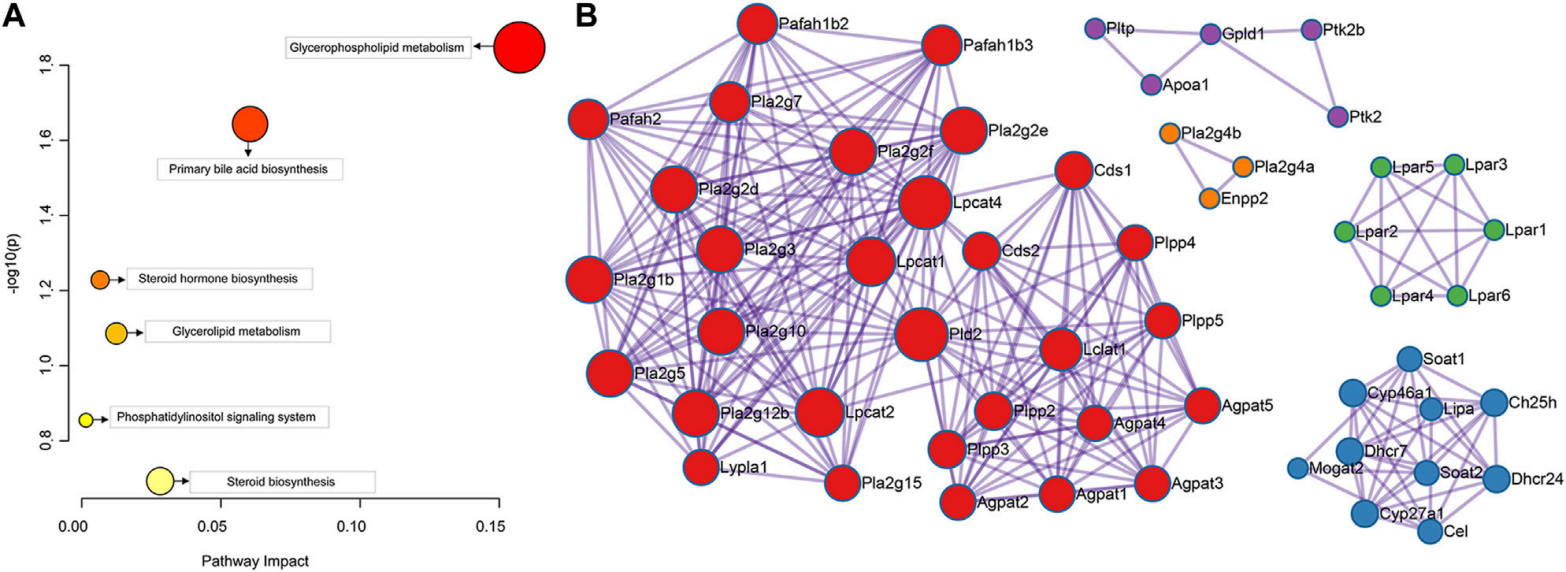
Pathway enrichment analysis of the serum differential biomarker **(A)** and the target screening of FXR deficiency-induced intrahepatic cholestasis **(B)**.

The results showed that 8 serum differential biomarkers related to FXR deficiency-induced intrahepatic cholestasis were enriched to 6 metabolic pathways (Figure 6A; Table 2), mainly glycophoric metabolism and primary bile acid biosynthesis. This result is consistent with the known function of FXR. To further reveal the upstream signals of the differential biomarkers related to FXR deficiency-induced intrahepatic cholestasis, the relevant targets of the above 8 differential metabolic markers were collected. As shown in Figure 6B, 131 targets have been collected and most targets can regulate each other.

**TABLE 2:** Metabolic-related pathways enriched by serum differential biomarker of FXR deficiency-induced intrahepatic cholestasis.

Pathway name	Match status	p	-log(p)	Impact
Glycerophospholipid metabolism	2/36	0.014215	1.8473	0.15723
Primary bile acid biosynthesis	2/46	0.022735	1.6433	0.06048
Steroid hormone biosynthesis	2/77	0.059138	1.2281	0.00662
Glycerolipid metabolism	1/16	0.082083	1.0857	0.01246
Phosphatidylinositol signaling system	1/28	0.13972	0.85476	0.00152
Steroid biosynthesis	1/42	0.20293	0.69265	0.0282

**FIGURE 7:**
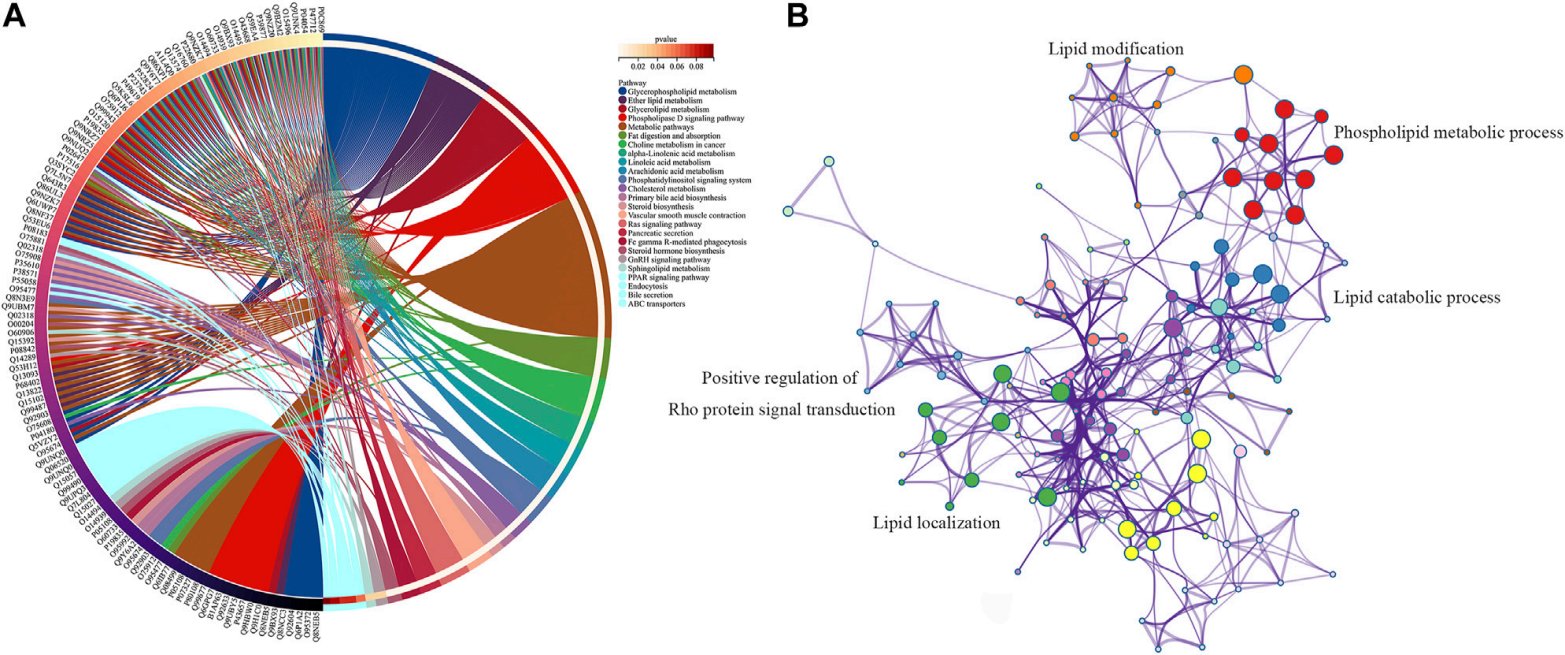
KEGG **(A)** and GO **(B)** enrichment analysis of the upstream targets of the biomarkers of FXR deficiency-induced intrahepatic cholestasis.

To investigate the signal pathway regulated by FXR in causing intrahepatic cholestasis, KEGG and GO enrichment analyses were performed. As we expected, the KEGG enrichment analysis results showed that FXR knockout caused bile acid metabolisms and its upstream lipid metabolism-related signal pathways, such as bile secret, primary bile acid biosynthesis, ABC transporters, glycerophospholipid metabolism, and steroid hormone biosynthesis. In addition, other signals were also enriched, such as the GnRH signaling pathway (*p* = 1.36 × 10^−4^) and Fc gamma R-mediated phase (*p* = 0.02) signals (Figure 7A and Supplementary Table S1). The GO enrichment analysis results showed that the targets were mainly enriched in lipid metabolism (Figure 7B).

### 3.6 Effect of FXR knockout on intestinal flora in mice

**FIGURE 8:**
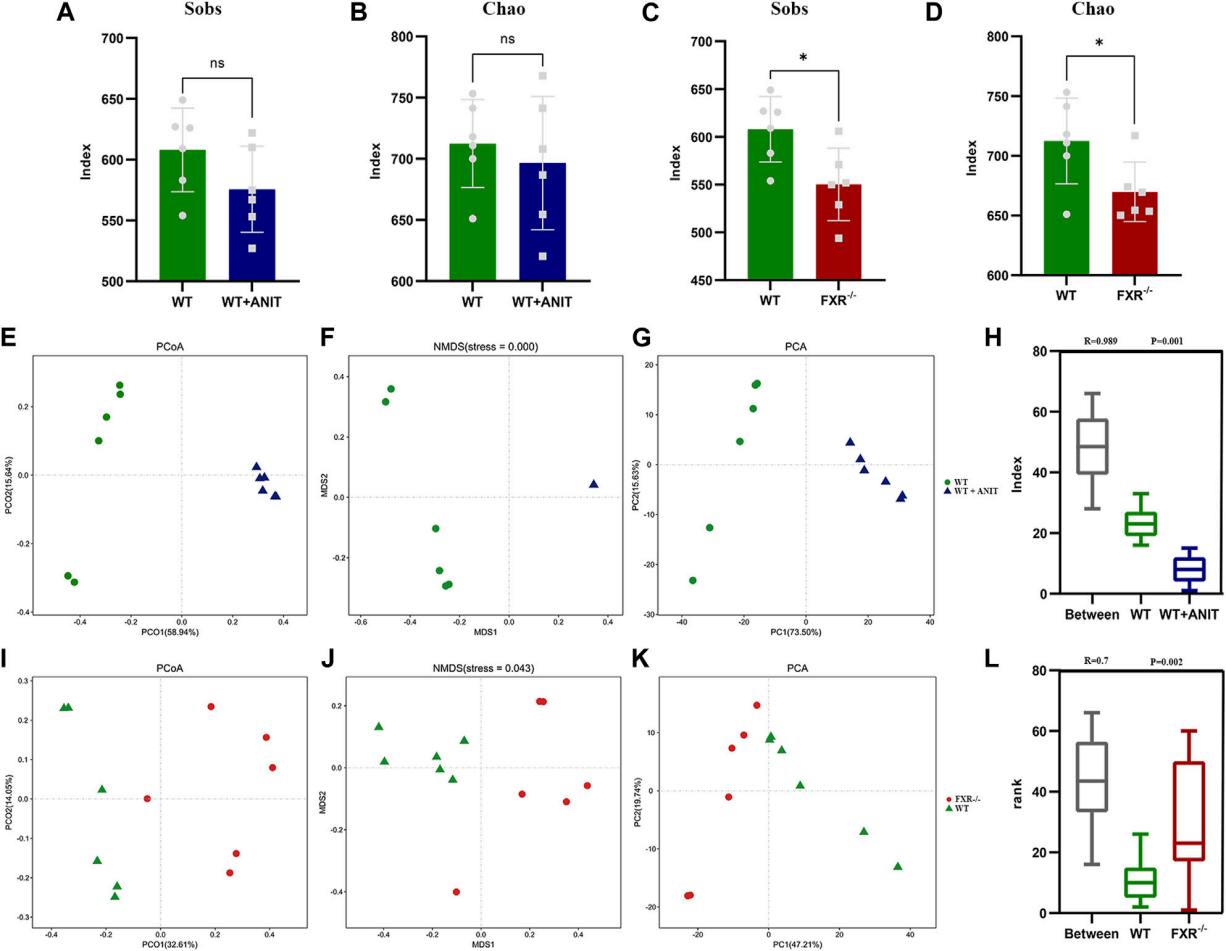
The changes of specific intestinal bacteria in mice. **(A, B)**: Sobs and Chao indices in the WT and WT + ANIT groups. **(C, D)**: Sobs and Chao indices in the WT and FXR^−/−^ groups. **(E-H)**: WT, WT + ANIT group microbiota unweighted PCoA, NMDS, PCA, and Anosim analysis chart. **(I-L)**: WT, FXR^−/−^ group microbiota unweighted PCoA, ANMDSD, PCA, and Anosim analysis chart. *p < 0.05.

Alpha diversity analysis reflects the species abundance and species diversity. Alpha diversity was presented as the index of Chao and Sobs. The results showed that the Sobs and Chao values of the WT + ANIT group and FXR^−/−^ group (*p* < 0.05) were both downregulated compared with WT mice (Figures 8A–D). The results of PCoA, NMDSD, and PCA showed that the microbial community structure was significantly different between the WT and WT + ANIT (Figures 8E–H), as well as WT and FXR^−/−^ (Figures 8I–L). Box diagram analysis and Anosim test results showed a significant difference between the WT and WT + ANIT groups (Figure 8H), as well as WT and FXR^−/−^ groups (Figure 8L).

**FIGURE 9:**
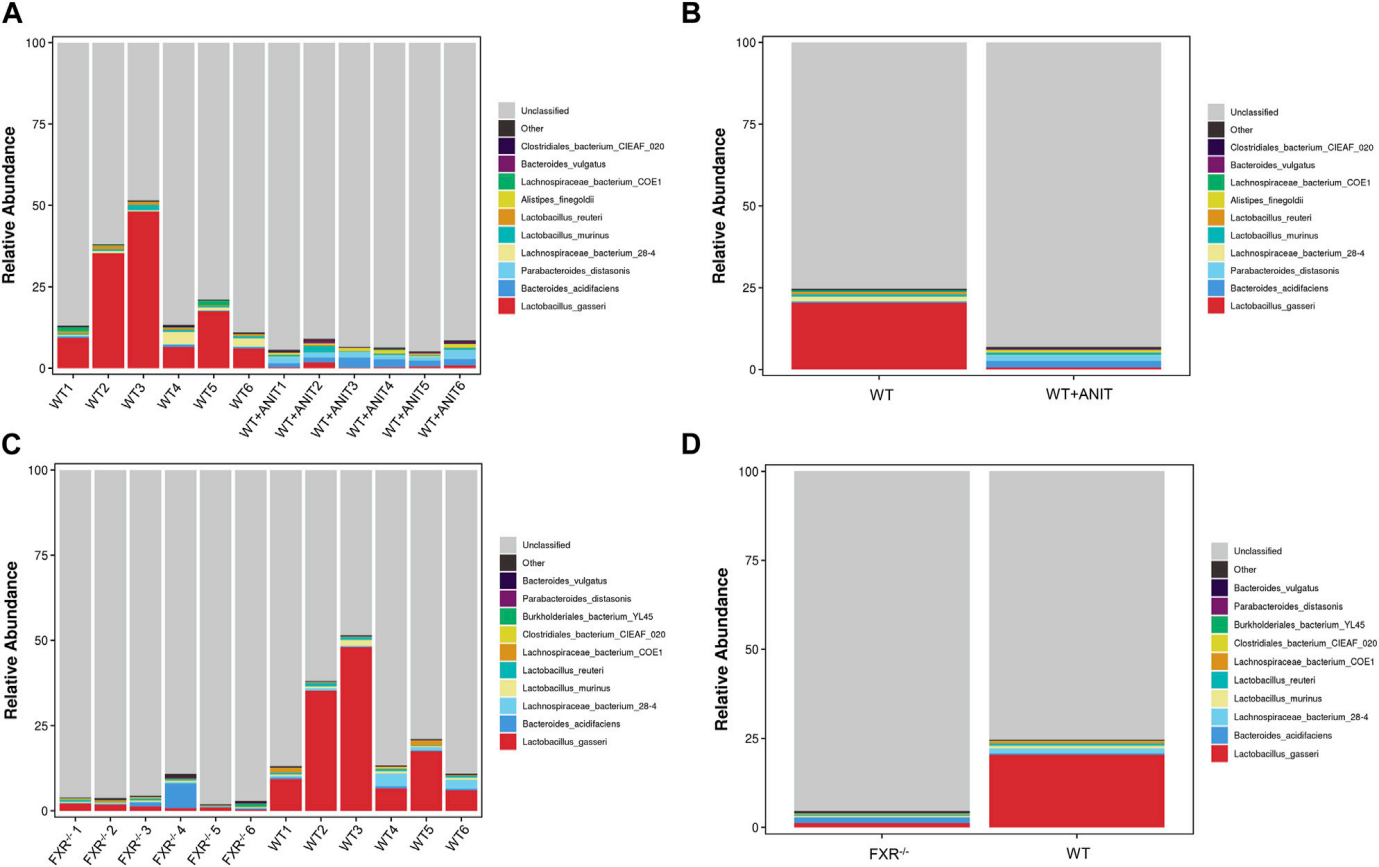
Relative abundance of intestinal flora at the genus, phylum, and species level. Compared with the WT group, the relative abundance of the intestinal flora at the species level in the WT + ANIT group **(A, B)**, and in the FXR^−/−^ group **(C, D)**.

**FIGURE 10:**
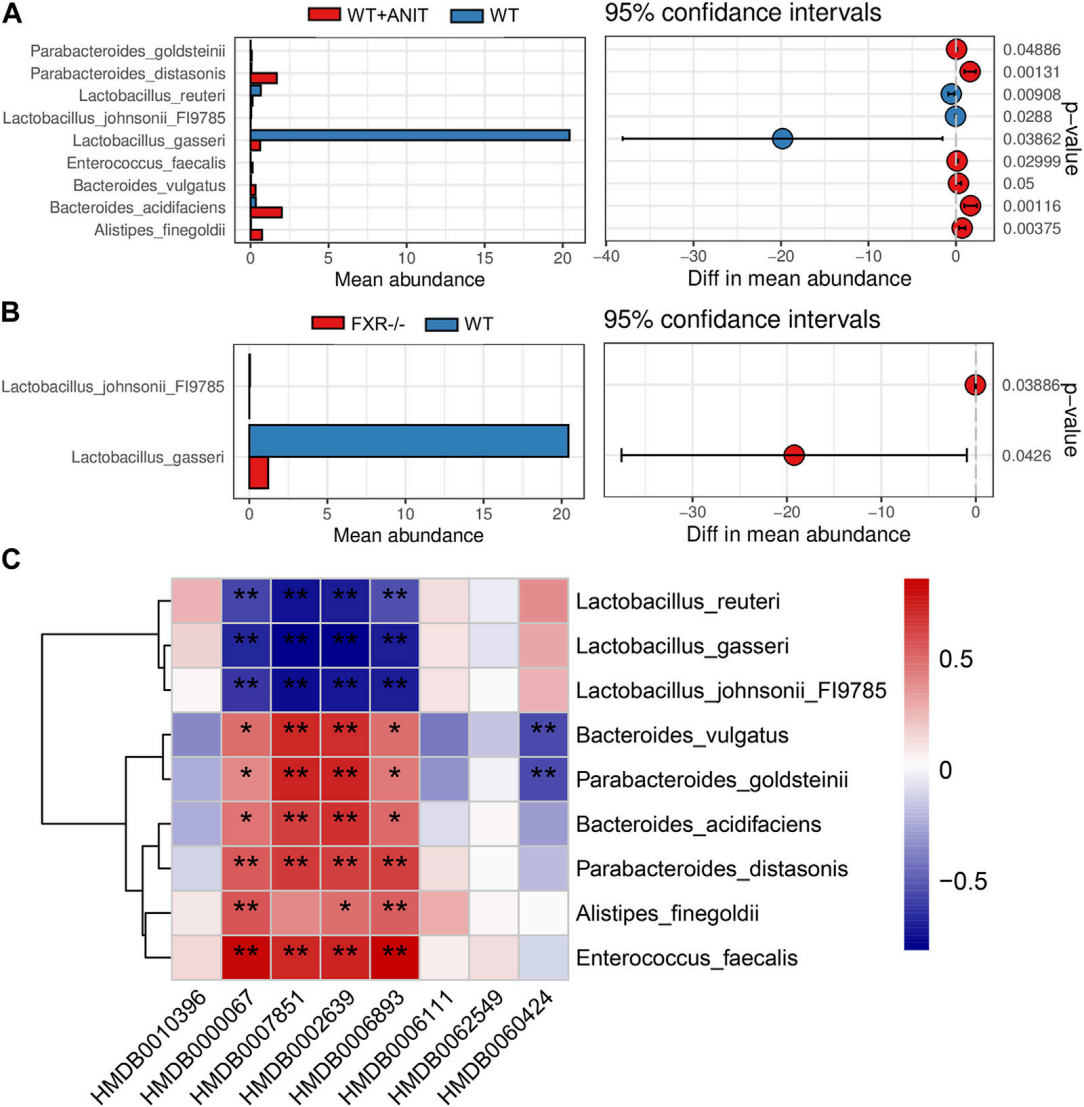
Functional enrichment analysis of mouse gut microbiota. **(A)** The differential flora in the WT + ANIT group compared with the WT group. **(B)** The differential flora in the WT + ANIT group compared with the FXR^−/−^ group. **(C)** Correlation analysis of differential bacteria species and differential metabolites. *p < 0.05, **p < 0.01.

The relative abundance of microbial species between the WT and WT + ANIT groups, and between the WT and FXR^−/−^ groups at genus, phylum, and species levels was calculated and depicted by stacked bar plots (Figure 10). At the genus level, compared with the WT group, the relative abundance of *Escherichia-Shigella* in the WT + ANIT group (Supplementary Figures S3A, B) and *Akkermansia* in the FXR^−/−^ group increased significantly, while the relative abundance of *Lactobacillus* in the WT + ANIT and FXR^−/−^ groups was significantly lower (Supplementary Figures S3C, D). At the phylum level, the relative abundance of *Proteobacteria* in the WT + ANIT group (Supplementary Figures S3E, F) and *Verrucomicrobia* in the FXR^−/−^ group (Supplementary Figures S3G, H) increased significantly compared with the WT group, while the relative abundance of *Firmicutes* in the WT + ANIT and FXR^−/−^ groups was significantly lower. At the species level, the relative abundance of *Parabacteroides_goldsteinii*, *Parabacteroides_distasonis*, *Enterococcus_faecalis*, *Bacteroides_vulgatus*, *Bacteroides_acidifaciens* and *Alistipes_finegoldii* in the WT + ANIT group (Figures 9A, B) increased significantly compared with WT group, while the relative abundance of *Lactobacillus_reuteri* and *Lactobacillus_johnsonii_FI9785* in the WT + ANIT and *Lactobacillus_johnsonii_FI9785* and *Lactobacillus_gasseri* in the FXR^−/−^ group was significantly lower (Figures 9A–D).

### 3.7 Functional enrichment analysis of differential intestinal flora

Compared with the WT group, the main signals enriched by different bacterial flora in the WT + ANIT group were inflammatory signals, bile acid metabolism signals, steroid metabolic signals, and fatty acid metabolism signals (Supplementary Figure S4). While, in the FXR^−/−^ group, the main signals enriched by different bacterial flora were bile acid metabolism, steroid metabolism, sphingomyelin metabolism, and amino acid metabolism, such as steroid hormone biosynthesis, sphingolipid, secondary bile acid biosynthesis, protein digestion and absorption, phenylalanine metabolism, NOD-like receptor signaling pathway, and beta-Alanine metabolism (Supplementary Figure S5).

### 3.8 Correlation analysis for differential biomarkers and microbes

A correlation heatmap was constructed to evaluate the covariation between altered gut microbiota species and differential biomarkers. As shown in Figure 10C, the differential gut microbiota and biomarkers were significantly correlated. The result demonstrated that the changes in these differential biomarkers may be associated with gut microbiota disruption. According to the above research, we found that the *Lactobacillus_johnsonii_*FI9785 and *Lactobacillus_gasseri were* significantly lower in FXR^−/−^ group. Correlation analysis results also found that *Lactobacillus_johnsonii_*FI9785 and *Lactobacillus_gasseri* were negatively correlated with the differential biomarkers (HMDB0000067, HMDB0007851, HMDB0002639, and HMDB0006893). This result proves that HMDB0000067, HMDB0007851, HMDB0002639, and HMDB0006893 play prominent roles in cholestasis caused by FXR knockout which is associated with *Lactobacillus_johnsonii_*FI9785 and *Lactobacillus_gasseri*.

## 4 Discussion

Disturbances of the gut–liver axis are associated with the pathogenesis of various liver diseases (Wiest et al., 2017). Pathological bacterial translocation from the intestine into the liver and systemic circulation is a risk factor for the pro-inflammatory response which can cause liver damage (Wiest & Garcia-Tsao, 2005; Jalan et al., 2014). FXR is a key target in the enterohepatic circulation of bile acids and has become an important drug target in the treatment of liver disease. Studies have found that gut-specific FXR agonism is a new method for treating obesity and metabolic syndrome (Fang et al., 2015). FXR agonist MET409 can significantly reduce liver fat after 12 weeks of treatment in NASH patients (Harrison et al., 2021). OCA treatment can reduce serum ALP in patients with PSC (Kowdley et al., 2020). However, the key role FXR plays in the metabolic system has not yet been systematically revealed.

Our current research finds that FXR^−/−^ can cause the spontaneous formation of intrahepatic cholestasis in mice. The gallbladder of FXR^−/−^ mice were significantly enlarged, inflammatory cells in liver tissue were significantly infiltrated, and liver cell nuclei were partially dissolved. The villi of ileal tissue are exfoliated, sparse, and low. Metabolomics is a high throughput detection method in comprehensively understanding the molecular mechanism of disease and the response of metabolic pathways to disturbances. It has been widely used to study metabolic product changes in pathological and medically treated subjects (Fillet & Frederich, 2015). Therefore, in this study, untargeted metabolomics is carried out to discover biomarkers associated with FXR deficiency-induced intrahepatic cholestasis. Eight differential biomarkers were found to be closely related to FXR deficiency-induced intrahepatic cholestasis by applying PCA and OPLS-DA analysis combined with multiple filter parameters. These differential biomarkers are mainly involved in primary bile acid metabolism, glycerides metabolism, and steroid metabolism pathways. Moreover, the upstream regulatory targets of these differential biomarkers are mainly enriched in bill secret, primary bill acid biosynthesis, ABC transporters, glycerophoric metabolism, steroid hormone biosynthesis, etc., which is consistent with the FXR function discovered by previous studies (Gonzalez, 2012; Xiao-Rong et al., 2021; Garrido et al., 2022).

Gut microbiota dysbiosis is closely related to the pathogenesis of intrahepatic cholestasis (Tang et al., 2023). Therefore, this study further analyzed the correlation between intestinal bacteria and FXR deficiency-induced intrahepatic cholestasis by the 16s rRNA gene sequencing. Our findings proved that the Sobs and Chao indices of the intestinal bacteria in the FXR^−/−^ mice were significantly decreased. Although ANIT caused significant cholestasis in mice, it has no significant effect on the Sobs and Chao indices of intestinal bacteria in WT mice. This result indicates that FXR^−/−^ has a greater impact on intestinal flora than ANIT in cholestatic mice, although both FXR knockout and ANIT can cause intrahepatic cholestasis.

FXR knockout caused the relative abundance of *Lactobacillus*_ *johnsonii*_ FI9785 and *Lactobacillus*_*gasseri* to decrease significantly. Similarly, the relative abundance of *Lactobacillus*_ *johnsonii*_ FI9785 was decreased significantly in the intrahepatic cholestasis induced by ANIT. This discovery suggests a clear correlation between *Lactobacillus*_ *johnsonii*_ FI9785 with the cholestasis caused by FXR deficiency. The differential intestinal bacteria in cholestatic mice caused by FXR knockout were mainly associated with bile acid metabolism, steroid metabolism, sphingomyelin metabolism, and amino acid metabolism. This result is consistent with the metabolic and KEGG signals of the differential metabolites associated with FXR deficiency-induced intrahepatic cholestasis. The correlation analysis for differential biomarkers and microbes found that *Lactobacillus_johnsonii_*FI9785 were negatively correlated with the differential biomarkers [cholesterol, LysoPA(0:0/18:1(9Z)], Sulfolithocholylglycine and 3a,7a-Dihydroxy-5b-cholestane) associated with the pathogenesis and progression of cholestasis caused by FXR knockout.

In the present study, FXR^−/−^ mice and a mouse model of cholestasis were used to comprehensively reveal the metabolic characteristics of mice in which FXR participates by detecting serum differential biomarkers and differential intestinal bacteria. We found that specific gut bacteria are closely related to FXR function in intrahepatic cholestasis. This study provides insights into the pathogenesis of FXR participating in intrahepatic cholestasis.

## Data Availability

The datasets presented in this study can be found in online repositories. The names of the repository/repositories and accession number(s) can be found below: https://www.ncbi.nlm.nih.gov/; PRJNA962200.
