# Supplementary figures and images for: Alterations in the gut microbiota and serum metabolomics of spontaneous cholestasis caused by loss of FXR signal in mice
Source: Front Pharmacol. 2023 May 22;14:1197847. doi: 10.3389/fphar.2023.1197847 (PMC10239812; doi:10.3389/fphar.2023.1197847)

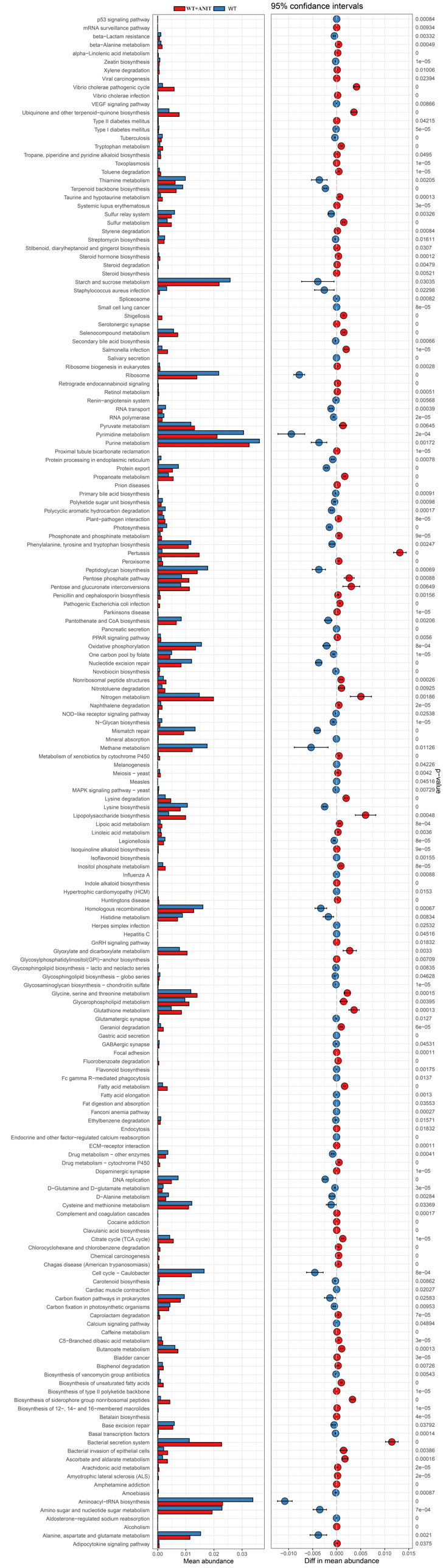

Supplement: Supplementary file 2 [file Image4.TIF]

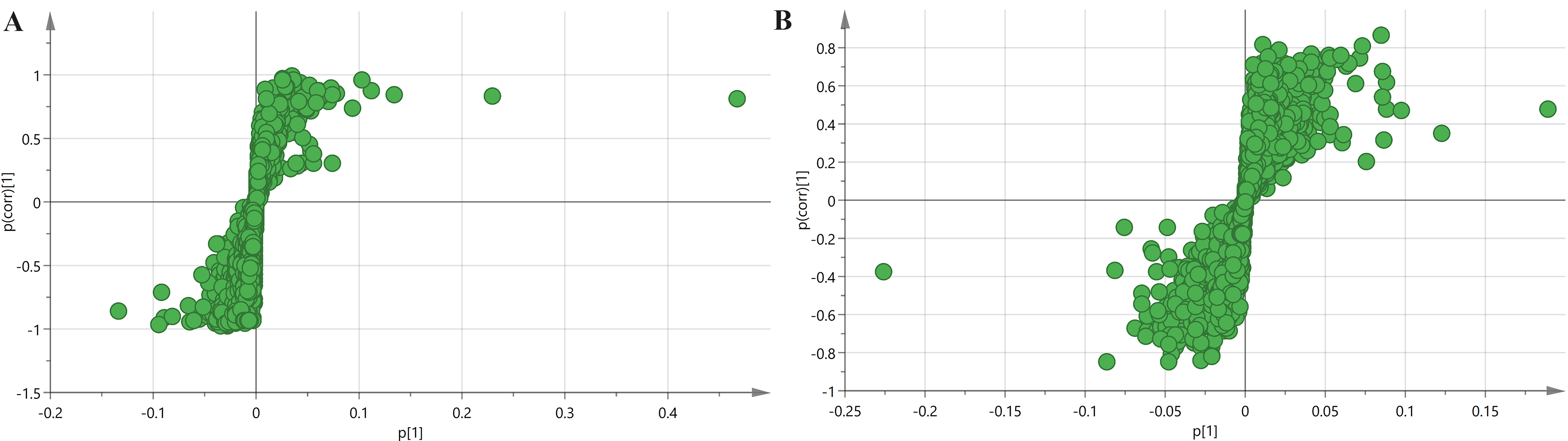

Supplement: Supplementary file 3 [file Image2.TIF]

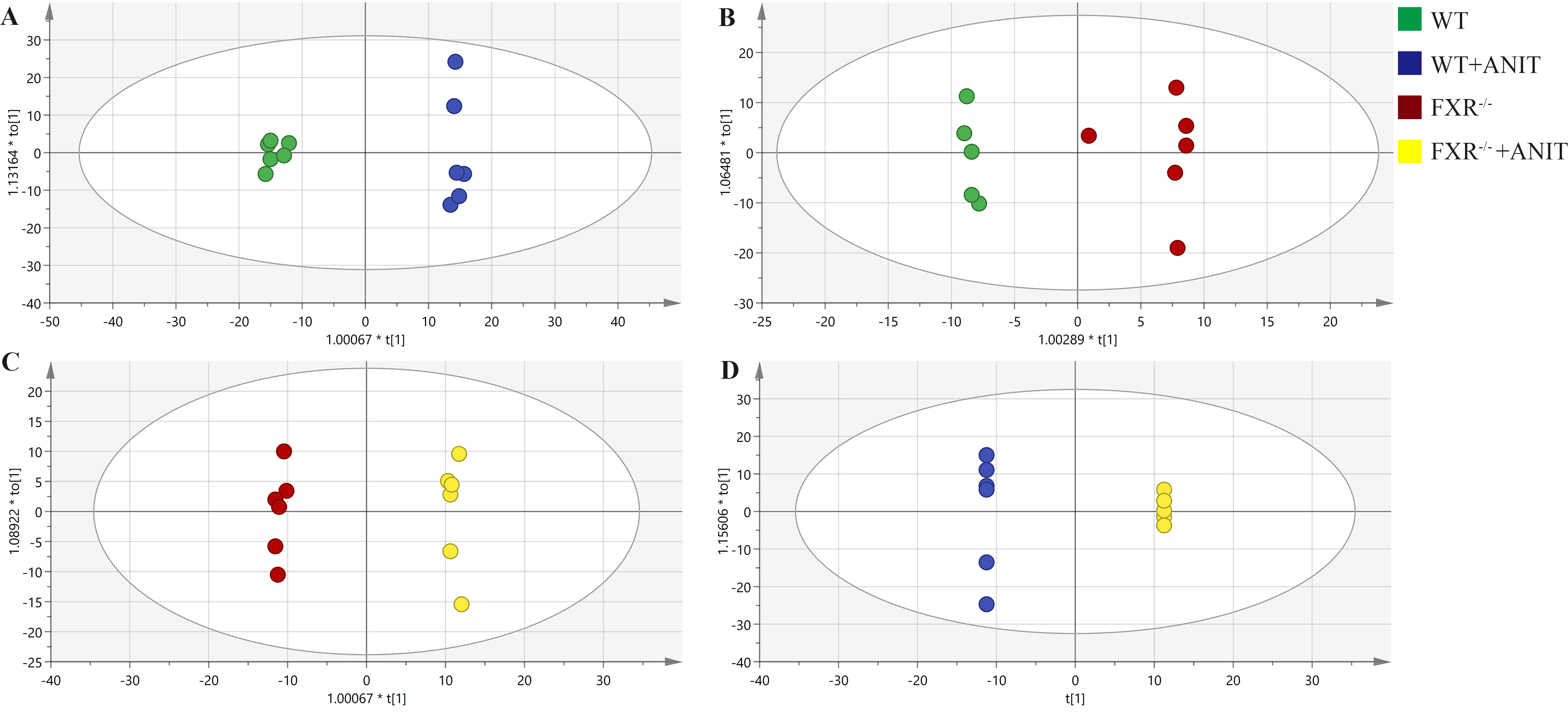

Supplement: Supplementary file 4 [file Image1.TIF]

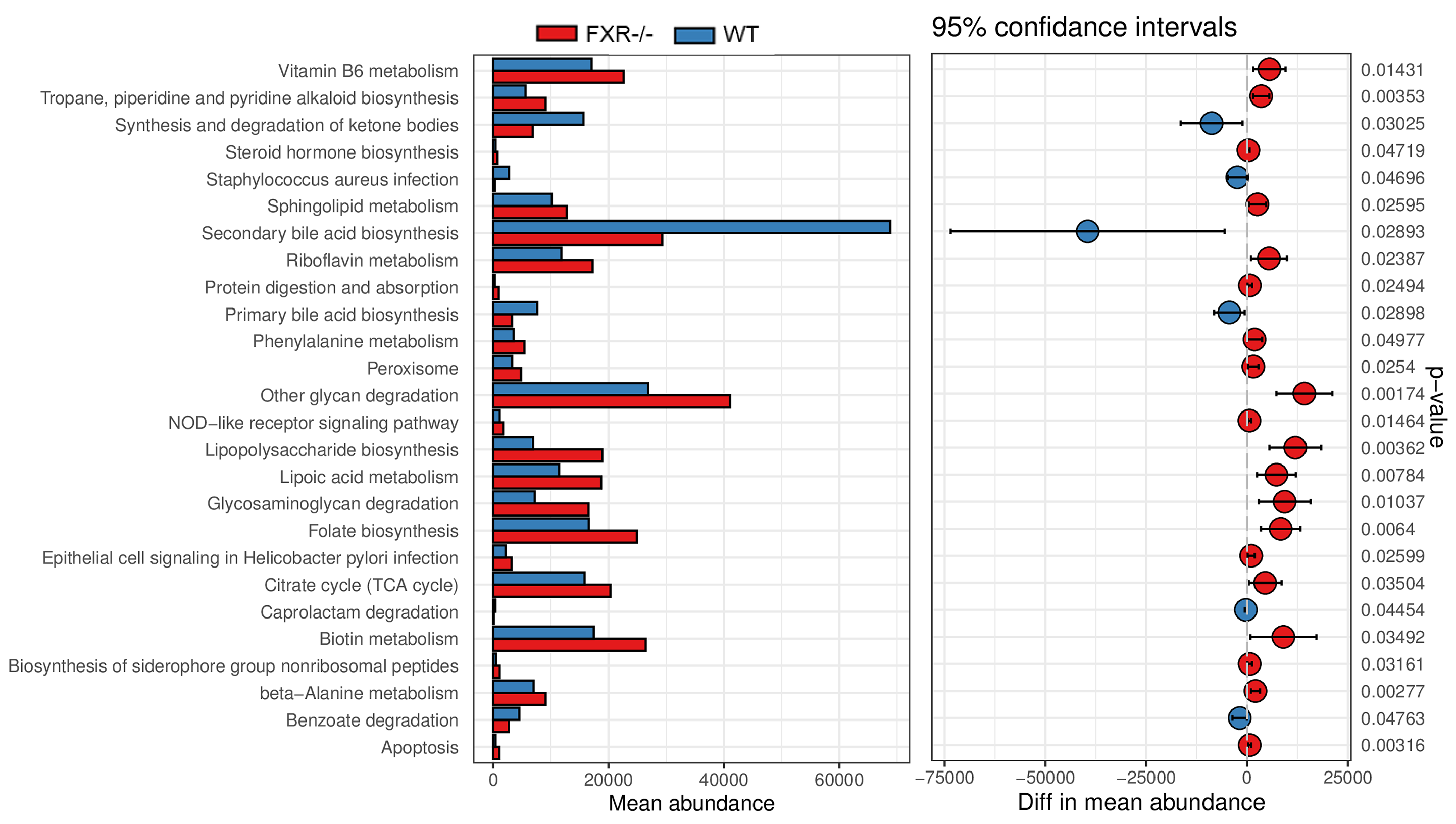

Supplement: Supplementary file 5 [file Image5.TIF]
